# Isopropyl 2,2-bis­(4-bromo­phen­yl)-2-hy­droxy­acetate

**DOI:** 10.1107/S1600536812044571

**Published:** 2012-11-03

**Authors:** Graham Smith

**Affiliations:** a Science and Engineering Faculty, Queensland University of Technology, GPO Box 2434, Brisbane, Queensland 4001, Australia

## Abstract

The title compound, C_17_H_16_Br_2_O_3_, which is a restricted commercial acaricide (common name bromo­propyl­ate), has two independent and conformationally similar mol­ecules in the asymmetric unit [dihedral angles between the planes of the two phenyl rings = 68.7 (4) and 77.4 (5)°]. The C atoms of the isopropyl group of one of the mol­ecules are disordered over two sites with occupancies of 0.638 (16) and 0.362 (16). Minor non-merohedral twinning was also present in the crystal. Inter­molecular O—H⋯O hydrogen-bonding inter­actions involving the hy­droxy groups and carboxyl O-atom acceptors give separate centrosymmetric homodimers through cyclic hydrogen-bonding motifs [graph set *R*_2_^2^(10)].

## Related literature

For background information on bromo­propyl­ate, see: O’Neil (2001 ▶). For the structures of benzilic acid and an analogous benzilate ester, see: Qui *et al.* (2007 ▶); Fu *et al.* (2006 ▶). For graph-set analysis, see: Etter *et al.* (1990 ▶).
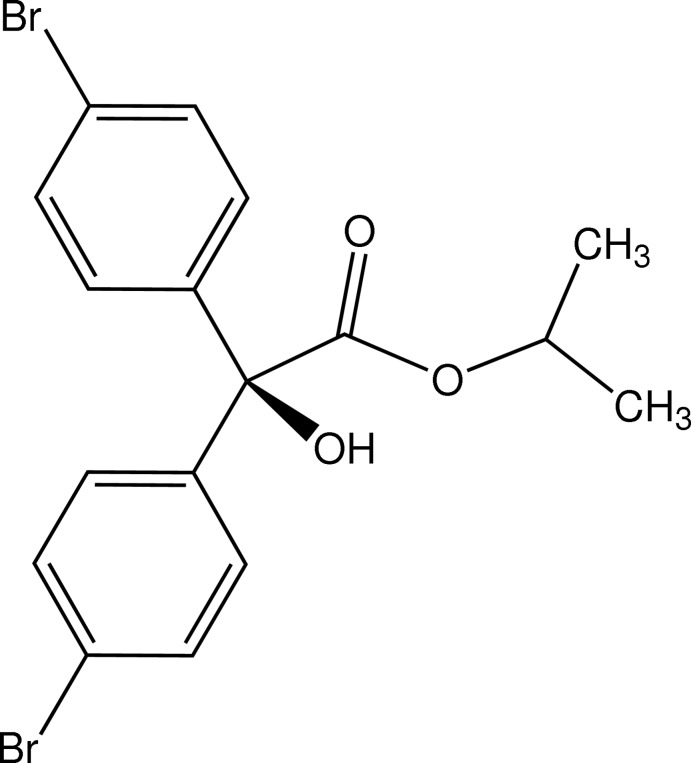


## Experimental

### 

#### Crystal data

C_17_H_16_Br_2_O_3_
*M* *_r_* = 428.10
Triclinic, 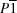

*a* = 10.2036 (6) Å
*b* = 10.2166 (6) Å
*c* = 17.6687 (13) Å
α = 83.775 (5)°
β = 73.346 (6)°
γ = 72.937 (5)°
*V* = 1686.4 (2) Å^3^
*Z* = 4
Mo *K*α radiation
μ = 4.82 mm^−1^
*T* = 200 K
0.30 × 0.20 × 0.12 mm

#### Data collection

Oxford Diffraction Gemini-S CCD-detector diffractometer
Absorption correction: multi-scan (*CrysAlis PRO*; Agilent, 2012 ▶) *T*_min_ = 0.581, *T*_max_ = 0.980
21160 measured reflections
6627 independent reflections
5435 reflections with *I* > 2σ(*I*)
*R*_int_ = 0.048

#### Refinement

*R*[*F* ^2^ > 2σ(*F* ^2^)] = 0.055
*wR*(*F* ^2^) = 0.156
*S* = 1.08
6627 reflections
408 parameters
H-atom parameters constrained
Δρ_max_ = 1.20 e Å^−3^
Δρ_min_ = −1.14 e Å^−3^

### 

Data collection: *CrysAlis PRO* (Agilent, 2012 ▶); cell refinement: *CrysAlis PRO*; data reduction: *CrysAlis PRO*; program(s) used to solve structure: *SIR92* (Altomare *et al.*, 1993 ▶); program(s) used to refine structure: *SHELXL97* (Sheldrick, 2008 ▶) within *WinGX* (Farrugia, 1999 ▶); molecular graphics: *PLATON* (Spek, 2009 ▶); software used to prepare material for publication: *PLATON*.

## Supplementary Material

Crystal structure: contains datablock(s) global, I. DOI: 10.1107/S1600536812044571/sj5277sup1.cif

Structure factors: contains datablock(s) I. DOI: 10.1107/S1600536812044571/sj5277Isup2.hkl

Supplementary material file. DOI: 10.1107/S1600536812044571/sj5277Isup3.cml

Additional supplementary materials:  crystallographic information; 3D view; checkCIF report

## Figures and Tables

**Table 1:** Hydrogen-bond geometry (Å, °)

*D*—H⋯*A*	*D*—H	H⋯*A*	*D*⋯*A*	*D*—H⋯*A*
O11*A*—H11*A*⋯O22*A* ^i^	0.94	1.99	2.866 (6)	154
O11*C*—H11*C*⋯O22*C* ^ii^	0.82	2.17	2.828 (6)	137

Symmetry codes: (i) 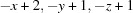; (ii) 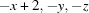.

**Fig. 1.**
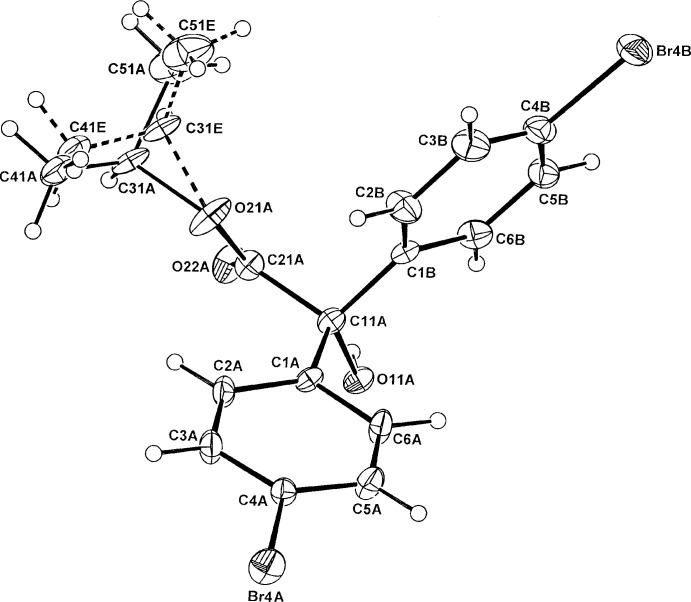
Molecular conformation and atom numbering scheme for molecule 1 of the two independent molecules in the asymmetric unit of the title compound, including the C atoms (C31E, C41E, C51E) of the disordered isopropyl group. Displacement ellipsoids are drawn at the 40% probability level.

**Fig. 2.**
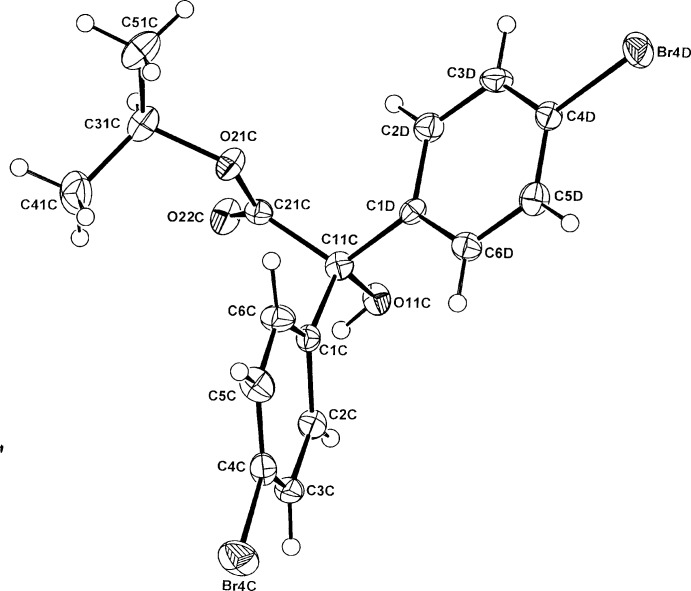
Molecular conformation and atom numbering scheme for the second molecule in the asymmetric unit of the title compound, with displacement ellipsoids drawn at the 40% probability level.

**Fig. 3.**
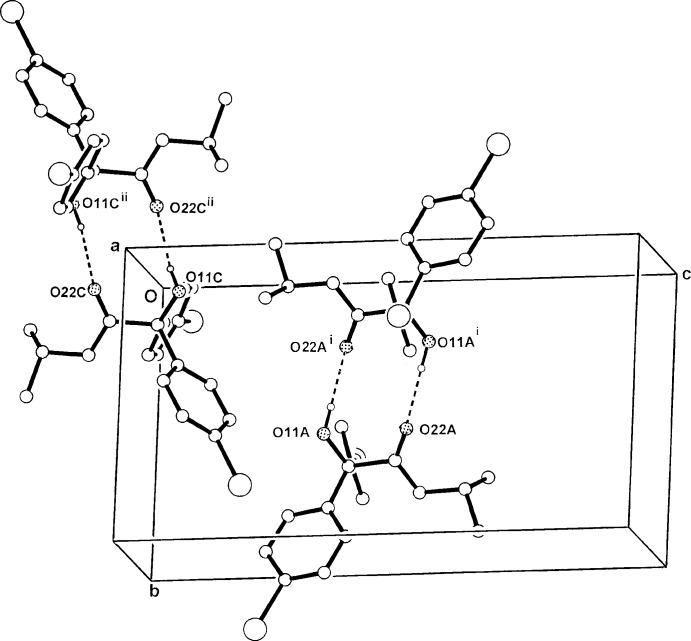
A perspective view of the two hydrogen-bonded homodimeric units in the unit cell, viewed along a. The minor disorder component of the isopropyl group of the first molecule is not shown.

## supplementary crystallographic information

### Comment

The title compound C_17_H_16_Br_2_O_3_, (common name bromopropylate) is the isopropyl ester of 4,4'-dibromobenzilic acid and is available for limited commercial use as a contact acaricide (Acarol, Neorol) (O'Neil, 2001). The structure of benzilic acid (Qui *et al.*, 2007) and its 1-methylpiperidin-4-yl ester (Fu *et al.*, 2006) have previously been reported.

In the structure of the title compound there are two independent and conformationally similar molecules in the asymmetric unit (Figs. 1, 2). The dihedral angles between the planes of the two phenyl rings in these molecules are 68.7 (4) and 77.4 (5)°. However, in one of the molecules the isopropyl group is disordered over two separate sites [C31*A*, C41*A*, C51*A* [S.O.F = 0.638 (16)] and C31*E*, C41*E*, C51*E* [S.O.F. = 0.362 (16)]. Minor non-merohedral twinning was also present in the crystal. Intermolecular hydrogen-bonding interactions involving the hydroxy groups and carboxyl O-atom acceptors (Table 1) give separate centrosymmetric homodimers through cyclic motifs [graph set *R*^2^_2_(10) (Etter *et al.*, 1990)] (Fig. 3). Other intramolecular aromatic C—H···O interactions are also present in the dimers as well as relatively short intermolecular Br···O interactions [Br4*B*···O11*A*^iii^, 3.348 (4) Å; Br4*C*···O11*C*^iii^, 3.389 (4) Å [for symmetry code (iii), *x*-1, *y*, *z*].

### Experimental

The title compound bromopropylate was obtained as an analytical reference standard from the U.S. Environmental Protection Agency. Colourless crystal plates suitable for X-ray analysis were obtained by room temperature evaporation of a solution in ethanol.

### Refinement

Hydroxyl H-atoms were located in a difference-Fourier synthesis but were subsequently allowed to ride in the refinement with *U*_iso_(H) = 1.2*U*_eq_(O). Other H-atoms were included in the refinement at calculated positions [C—H = 0.93 Å (aromatic), 0.97 Å (methine) and 0.96 Å (methyl), with *U*_iso_(H) = 1.2*U*_eq_(aromatic or methine C) or 1.5*U*_eq_(methyl C)], also using a riding-model approximation. Disorder was present in the C-atoms of the isopropyl group of one of the molecules and the occupancies were determined as 0.638 (16) [atoms C31*A*, C41*A*, C51*A*], and 0.362 (16) [atoms C31*E*, C41*E*, C51*E*]. Minor non-merohedral twinning was identified and handled in the refinement (BASF = 0.0779). The maximum difference electron density peak was 1.195 Å^-3^ (0.90 Å from Br4*B*).

### Crystal data

**Table:**

C_17_H_16_Br_2_O_3_	*Z* = 4
*M**_r_* = 428.10	*F*(000) = 848
Triclinic, *P*1	*D*_x_ = 1.686 Mg m^−^^3^
Hall symbol: -P 1	Melting point: 350 K
*a* = 10.2036 (6) Å	Mo *K*α radiation, λ = 0.71073 Å
*b* = 10.2166 (6) Å	Cell parameters from 5077 reflections
*c* = 17.6687 (13) Å	θ = 3.1–28.9°
α = 83.775 (5)°	µ = 4.82 mm^−^^1^
β = 73.346 (6)°	*T* = 200 K
γ = 72.937 (5)°	Block, colourless
*V* = 1686.4 (2) Å^3^	0.30 × 0.20 × 0.12 mm

### Data collection

**Table:**

Oxford Diffraction Gemini-S CCD-detector diffractometer	6627 independent reflections
Radiation source: Enhance (Mo) X-ray source	5435 reflections with *I* > 2σ(*I*)
Graphite monochromator	*R*_int_ = 0.048
Detector resolution: 16.077 pixels mm^-1^	θ_max_ = 26.0°, θ_min_ = 3.1°
ω scans	*h* = −12→12
Absorption correction: multi-scan (*CrysAlis PRO*; Agilent, 2012)	*k* = −12→12
*T*_min_ = 0.581, *T*_max_ = 0.980	*l* = −21→21
21160 measured reflections	

### Refinement

**Table:**

Refinement on *F*^2^	Primary atom site location: structure-invariant direct methods
Least-squares matrix: full	Secondary atom site location: difference Fourier map
*R*[*F*^2^ > 2σ(*F*^2^)] = 0.055	Hydrogen site location: inferred from neighbouring sites
*wR*(*F*^2^) = 0.156	H-atom parameters constrained
*S* = 1.08	*w* = 1/[σ^2^(*F*_o_^2^) + (0.0571*P*)^2^ + 8.7092*P*] where *P* = (*F*_o_^2^ + 2*F*_c_^2^)/3
6627 reflections	(Δ/σ)_max_ = 0.002
408 parameters	Δρ_max_ = 1.20 e Å^−^^3^
0 restraints	Δρ_min_ = −1.14 e Å^−^^3^

### Special details

**Table:**

Geometry. Bond distances, angles *etc*. have been calculated using the rounded fractional coordinates. All su's are estimated from the variances of the (full) variance-covariance matrix. The cell e.s.d.'s are taken into account in the estimation of distances, angles and torsion angles
Refinement. Refinement of *F*^2^ against ALL reflections. The weighted *R*-factor *wR* and goodness of fit *S* are based on *F*^2^, conventional *R*-factors *R* are based on *F*, with *F* set to zero for negative *F*^2^. The threshold expression of *F*^2^ > σ(*F*^2^) is used only for calculating *R*-factors(gt) *etc*. and is not relevant to the choice of reflections for refinement. *R*-factors based on *F*^2^ are statistically about twice as large as those based on *F*, and *R*- factors based on ALL data will be even larger.

### Fractional atomic coordinates and isotropic or equivalent isotropic displacement parameters (Å^2^)

**Table:**

	*x*	*y*	*z*	*U*_iso_*/*U*_eq_	Occ. (<1)
Br4A	0.89518 (9)	1.30205 (7)	0.27385 (4)	0.0459 (3)	
Br4B	0.30004 (9)	0.64421 (10)	0.40830 (5)	0.0564 (3)	
O11A	0.9752 (4)	0.6496 (4)	0.3992 (2)	0.0296 (12)	
O21A	0.7448 (6)	0.8256 (5)	0.5730 (3)	0.0483 (18)	
O22A	0.9228 (6)	0.6320 (5)	0.5558 (3)	0.0418 (17)	
C1A	0.8626 (6)	0.8855 (6)	0.4047 (3)	0.0237 (17)	
C1B	0.7154 (7)	0.7172 (6)	0.4347 (3)	0.0267 (17)	
C2A	0.8808 (7)	0.9874 (7)	0.4424 (4)	0.0324 (19)	
C2B	0.5880 (8)	0.8199 (7)	0.4497 (5)	0.043 (3)	
C3A	0.8907 (7)	1.1118 (7)	0.4037 (4)	0.034 (2)	
C3B	0.4654 (8)	0.7966 (8)	0.4439 (5)	0.045 (3)	
C4A	0.8842 (7)	1.1319 (6)	0.3272 (4)	0.0281 (17)	
C4B	0.4695 (8)	0.6706 (8)	0.4218 (4)	0.040 (3)	
C5A	0.8678 (8)	1.0319 (7)	0.2868 (4)	0.038 (2)	
C5B	0.5937 (8)	0.5666 (7)	0.4060 (4)	0.036 (2)	
C6A	0.8579 (8)	0.9096 (7)	0.3262 (4)	0.036 (2)	
C6B	0.7167 (8)	0.5900 (7)	0.4130 (4)	0.035 (2)	
C11A	0.8518 (6)	0.7449 (6)	0.4404 (4)	0.0260 (17)	
C21A	0.8464 (7)	0.7282 (6)	0.5296 (4)	0.0310 (19)	
C31A	0.7366 (17)	0.8166 (16)	0.6594 (8)	0.040 (4)	0.638 (16)
C41A	0.719 (3)	0.9601 (19)	0.6863 (12)	0.056 (6)	0.638 (16)
C51A	0.609 (2)	0.7606 (19)	0.6920 (10)	0.079 (7)	0.638 (16)
C51E	0.525 (4)	0.828 (4)	0.6798 (19)	0.079 (7)	0.362 (16)
C31E	0.675 (3)	0.802 (3)	0.6601 (17)	0.040 (4)	0.362 (16)
C41E	0.723 (5)	0.904 (4)	0.697 (2)	0.056 (6)	0.362 (16)
Br4C	0.18617 (7)	0.14249 (8)	0.07253 (5)	0.0447 (3)	
Br4D	0.66910 (9)	0.77309 (7)	0.21608 (4)	0.0419 (2)	
O11C	0.8480 (4)	0.1314 (4)	0.0961 (2)	0.0261 (12)	
O21C	0.8124 (4)	0.3361 (4)	−0.0781 (2)	0.0285 (12)	
O22C	0.9644 (5)	0.1328 (4)	−0.0599 (2)	0.0317 (12)	
C1C	0.6305 (6)	0.2050 (6)	0.0518 (3)	0.0208 (17)	
C1D	0.7444 (6)	0.3692 (6)	0.0908 (3)	0.0208 (17)	
C2C	0.5973 (7)	0.0877 (6)	0.0871 (3)	0.0260 (17)	
C2D	0.8500 (7)	0.4364 (6)	0.0735 (4)	0.0282 (17)	
C3C	0.4664 (7)	0.0675 (6)	0.0915 (4)	0.0288 (17)	
C3D	0.8284 (7)	0.5557 (6)	0.1114 (4)	0.0300 (17)	
C4C	0.3697 (7)	0.1655 (7)	0.0614 (4)	0.0302 (17)	
C4D	0.7019 (7)	0.6066 (6)	0.1669 (3)	0.0272 (19)	
C5C	0.4017 (7)	0.2818 (7)	0.0237 (4)	0.034 (2)	
C5D	0.5988 (7)	0.5403 (6)	0.1858 (4)	0.0301 (17)	
C6C	0.5316 (7)	0.3007 (6)	0.0177 (4)	0.0298 (19)	
C6D	0.6194 (7)	0.4215 (6)	0.1476 (4)	0.0296 (17)	
C11C	0.7712 (6)	0.2330 (6)	0.0516 (3)	0.0231 (17)	
C21C	0.8619 (6)	0.2286 (6)	−0.0351 (3)	0.0222 (17)	
C31C	0.8718 (7)	0.3287 (7)	−0.1646 (4)	0.0315 (19)	
C41C	0.8017 (9)	0.2421 (9)	−0.1959 (5)	0.050 (3)	
C51C	0.8390 (10)	0.4762 (8)	−0.1954 (4)	0.054 (3)	
H2A	0.88650	0.97300	0.49440	0.0390*	
H5A	0.86360	1.04660	0.23460	0.0460*	
H5B	0.59530	0.48160	0.39090	0.0430*	
H6A	0.84780	0.84100	0.29960	0.0430*	
H6B	0.80110	0.51980	0.40300	0.0420*	
H11A	0.99300	0.56760	0.42950	0.0440*	
H31A	0.82220	0.75160	0.66950	0.0480*	0.638 (16)
H41A	0.80580	0.98550	0.66400	0.0850*	0.638 (16)
H42A	0.69670	0.95970	0.74300	0.0850*	0.638 (16)
H43A	0.64300	1.02480	0.66900	0.0850*	0.638 (16)
H51A	0.62890	0.67200	0.67090	0.1190*	0.638 (16)
H52A	0.52790	0.82160	0.67720	0.1190*	0.638 (16)
H53A	0.58790	0.75290	0.74860	0.1190*	0.638 (16)
H2B	0.58560	0.90580	0.46400	0.0520*	
H3A	0.90180	1.18050	0.42980	0.0400*	
H3B	0.38020	0.86580	0.45490	0.0550*	
H31E	0.71740	0.70820	0.67680	0.0480*	0.362 (16)
H41E	0.82480	0.87560	0.68620	0.0850*	0.362 (16)
H42E	0.68060	0.90620	0.75260	0.0850*	0.362 (16)
H43E	0.69380	0.99360	0.67350	0.0850*	0.362 (16)
H51E	0.50110	0.75290	0.66420	0.1190*	0.362 (16)
H52E	0.48540	0.91050	0.65260	0.1190*	0.362 (16)
H53E	0.48630	0.83940	0.73580	0.1190*	0.362 (16)
H2C	0.66290	0.02170	0.10820	0.0310*	
H2D	0.93580	0.40110	0.03620	0.0340*	
H3C	0.44480	−0.01240	0.11480	0.0350*	
H3D	0.89900	0.60100	0.09940	0.0360*	
H5C	0.33570	0.34700	0.00250	0.0410*	
H5D	0.51450	0.57450	0.22430	0.0360*	
H6C	0.55480	0.37770	−0.00930	0.0350*	
H6D	0.54840	0.37670	0.16030	0.0350*	
H11C	0.86350	0.05570	0.07840	0.0390*	
H31C	0.97500	0.28740	−0.17760	0.0440*	
H41C	0.82590	0.15020	−0.17490	0.0750*	
H42C	0.83450	0.24110	−0.25250	0.0750*	
H43C	0.70030	0.28010	−0.18010	0.0750*	
H51C	0.88640	0.52610	−0.17430	0.0810*	
H52C	0.73820	0.51770	−0.17930	0.0810*	
H53C	0.87170	0.47810	−0.25200	0.0810*	

### Atomic displacement parameters (Å^2^)

**Table:**

	*U*^11^	*U*^22^	*U*^33^	*U*^12^	*U*^13^	*U*^23^
Br4A	0.0662 (5)	0.0316 (4)	0.0400 (4)	−0.0165 (3)	−0.0145 (4)	0.0082 (3)
Br4B	0.0428 (4)	0.0875 (7)	0.0494 (5)	−0.0334 (4)	−0.0163 (4)	0.0080 (4)
O11A	0.028 (2)	0.030 (2)	0.023 (2)	−0.0009 (18)	−0.0006 (18)	−0.0052 (17)
O21A	0.067 (4)	0.035 (3)	0.019 (2)	0.010 (2)	0.000 (2)	0.0008 (19)
O22A	0.054 (3)	0.034 (3)	0.033 (3)	0.002 (2)	−0.020 (2)	0.001 (2)
C1A	0.021 (3)	0.030 (3)	0.016 (3)	−0.003 (2)	−0.003 (2)	0.000 (2)
C1B	0.028 (3)	0.029 (3)	0.019 (3)	−0.007 (3)	−0.001 (3)	0.000 (2)
C2A	0.043 (4)	0.035 (3)	0.025 (3)	−0.014 (3)	−0.015 (3)	0.000 (3)
C2B	0.032 (4)	0.035 (4)	0.056 (5)	0.001 (3)	−0.007 (3)	−0.013 (3)
C3A	0.044 (4)	0.034 (4)	0.031 (3)	−0.017 (3)	−0.015 (3)	−0.003 (3)
C3B	0.026 (4)	0.054 (5)	0.049 (5)	−0.003 (3)	−0.005 (3)	−0.007 (4)
C4A	0.035 (3)	0.024 (3)	0.027 (3)	−0.009 (3)	−0.011 (3)	0.002 (2)
C4B	0.038 (4)	0.057 (5)	0.031 (4)	−0.024 (3)	−0.013 (3)	0.013 (3)
C5A	0.055 (5)	0.041 (4)	0.022 (3)	−0.014 (3)	−0.014 (3)	−0.001 (3)
C5B	0.043 (4)	0.033 (4)	0.037 (4)	−0.016 (3)	−0.015 (3)	0.004 (3)
C6A	0.058 (5)	0.032 (3)	0.023 (3)	−0.017 (3)	−0.015 (3)	−0.003 (3)
C6B	0.039 (4)	0.028 (3)	0.038 (4)	−0.011 (3)	−0.010 (3)	0.006 (3)
C11A	0.025 (3)	0.024 (3)	0.024 (3)	0.000 (2)	−0.005 (3)	−0.003 (2)
C21A	0.040 (4)	0.020 (3)	0.030 (3)	−0.006 (3)	−0.007 (3)	−0.001 (2)
C31A	0.041 (10)	0.043 (6)	0.018 (4)	−0.004 (7)	0.007 (7)	0.010 (4)
C41A	0.080 (8)	0.061 (14)	0.020 (7)	−0.016 (12)	−0.007 (6)	0.004 (9)
C51A	0.097 (15)	0.070 (12)	0.052 (8)	−0.030 (9)	0.012 (9)	0.011 (8)
C51E	0.097 (15)	0.070 (12)	0.052 (8)	−0.030 (9)	0.012 (9)	0.011 (8)
C31E	0.041 (10)	0.043 (6)	0.018 (4)	−0.004 (7)	0.007 (7)	0.010 (4)
C41E	0.080 (8)	0.061 (14)	0.020 (7)	−0.016 (12)	−0.007 (6)	0.004 (9)
Br4C	0.0290 (4)	0.0519 (5)	0.0557 (5)	−0.0159 (3)	−0.0076 (3)	−0.0092 (3)
Br4D	0.0655 (5)	0.0264 (3)	0.0359 (4)	−0.0129 (3)	−0.0150 (4)	−0.0055 (3)
O11C	0.030 (2)	0.022 (2)	0.026 (2)	−0.0013 (17)	−0.0136 (19)	0.0005 (16)
O21C	0.031 (2)	0.027 (2)	0.020 (2)	0.0001 (18)	−0.0045 (18)	0.0013 (17)
O22C	0.033 (2)	0.029 (2)	0.026 (2)	−0.0002 (19)	−0.0057 (19)	−0.0008 (18)
C1C	0.024 (3)	0.020 (3)	0.018 (3)	−0.005 (2)	−0.006 (2)	−0.001 (2)
C1D	0.022 (3)	0.021 (3)	0.018 (3)	−0.005 (2)	−0.005 (2)	0.002 (2)
C2C	0.030 (3)	0.020 (3)	0.024 (3)	−0.002 (2)	−0.006 (3)	−0.001 (2)
C2D	0.022 (3)	0.033 (3)	0.029 (3)	−0.008 (3)	−0.005 (3)	−0.002 (3)
C3C	0.035 (3)	0.023 (3)	0.028 (3)	−0.014 (3)	−0.001 (3)	−0.002 (2)
C3D	0.029 (3)	0.033 (3)	0.030 (3)	−0.015 (3)	−0.006 (3)	0.003 (3)
C4C	0.030 (3)	0.035 (3)	0.024 (3)	−0.008 (3)	−0.003 (3)	−0.008 (3)
C4D	0.042 (4)	0.017 (3)	0.022 (3)	−0.005 (3)	−0.011 (3)	0.000 (2)
C5C	0.028 (3)	0.036 (4)	0.042 (4)	−0.007 (3)	−0.018 (3)	0.000 (3)
C5D	0.023 (3)	0.034 (3)	0.029 (3)	−0.002 (3)	−0.004 (3)	−0.007 (3)
C6C	0.031 (3)	0.023 (3)	0.035 (4)	−0.008 (3)	−0.010 (3)	0.006 (2)
C6D	0.026 (3)	0.033 (3)	0.028 (3)	−0.009 (3)	−0.003 (3)	−0.003 (3)
C11C	0.024 (3)	0.020 (3)	0.024 (3)	−0.003 (2)	−0.009 (2)	0.003 (2)
C21C	0.022 (3)	0.019 (3)	0.026 (3)	−0.005 (2)	−0.008 (2)	0.000 (2)
C31C	0.029 (3)	0.038 (4)	0.021 (3)	−0.004 (3)	−0.003 (3)	0.002 (3)
C41C	0.059 (5)	0.060 (5)	0.038 (4)	−0.016 (4)	−0.022 (4)	−0.002 (4)
C51C	0.071 (6)	0.049 (5)	0.033 (4)	−0.014 (4)	−0.009 (4)	0.016 (3)

### Geometric parameters (Å, º)

**Table:**

Br4A—C4A	1.903 (6)	C41A—H41A	0.9600
Br4B—C4B	1.909 (9)	C41A—H42A	0.9600
Br4C—C4C	1.907 (8)	C41A—H43A	0.9600
Br4D—C4D	1.892 (6)	C41E—H41E	0.9600
O11A—C11A	1.404 (7)	C41E—H42E	0.9500
O21A—C31E	1.53 (3)	C41E—H43E	0.9600
O21A—C31A	1.499 (15)	C51A—H51A	0.9600
O21A—C21A	1.326 (8)	C51A—H53A	0.9600
O22A—C21A	1.203 (8)	C51A—H52A	0.9600
O11A—H11A	0.9400	C51E—H51E	0.9600
O11C—C11C	1.417 (7)	C51E—H53E	0.9600
O21C—C31C	1.475 (8)	C51E—H52E	0.9600
O21C—C21C	1.324 (7)	C1C—C2C	1.377 (8)
O22C—C21C	1.214 (7)	C1C—C6C	1.407 (9)
O11C—H11C	0.8200	C1C—C11C	1.543 (9)
C1A—C11A	1.526 (8)	C1D—C6D	1.379 (9)
C1A—C6A	1.394 (9)	C1D—C11C	1.539 (8)
C1A—C2A	1.377 (9)	C1D—C2D	1.388 (10)
C1B—C2B	1.387 (10)	C2C—C3C	1.390 (11)
C1B—C11A	1.531 (10)	C2D—C3D	1.385 (9)
C1B—C6B	1.390 (9)	C3C—C4C	1.365 (10)
C2A—C3A	1.393 (10)	C3D—C4D	1.375 (9)
C2B—C3B	1.373 (12)	C4C—C5C	1.376 (10)
C3A—C4A	1.363 (10)	C4D—C5D	1.358 (10)
C3B—C4B	1.371 (11)	C5C—C6C	1.368 (11)
C4A—C5A	1.381 (10)	C5D—C6D	1.386 (9)
C4B—C5B	1.373 (11)	C11C—C21C	1.544 (7)
C5A—C6A	1.379 (10)	C31C—C41C	1.518 (12)
C5B—C6B	1.385 (12)	C31C—C51C	1.520 (10)
C11A—C21A	1.554 (10)	C2C—H2C	0.9300
C31A—C51A	1.51 (3)	C2D—H2D	0.9300
C31A—C41A	1.54 (3)	C3C—H3C	0.9300
C31E—C41E	1.54 (5)	C3D—H3D	0.9300
C31E—C51E	1.42 (5)	C5C—H5C	0.9300
C2A—H2A	0.9300	C5D—H5D	0.9300
C2B—H2B	0.9300	C6C—H6C	0.9300
C3A—H3A	0.9300	C6D—H6D	0.9300
C3B—H3B	0.9300	C31C—H31C	0.9800
C5A—H5A	0.9300	C41C—H41C	0.9600
C5B—H5B	0.9300	C41C—H42C	0.9600
C6A—H6A	0.9300	C41C—H43C	0.9600
C6B—H6B	0.9300	C51C—H51C	0.9600
C31A—H31A	0.9800	C51C—H52C	0.9600
C31E—H31E	0.9800	C51C—H53C	0.9600
			
C21A—O21A—C31A	115.5 (8)	H52A—C51A—H53A	109.00
C21A—O21A—C31E	122.2 (12)	H51A—C51A—H52A	109.00
C11A—O11A—H11A	110.00	H51A—C51A—H53A	109.00
C21C—O21C—C31C	117.2 (4)	C31A—C51A—H51A	110.00
C11C—O11C—H11C	110.00	C31A—C51A—H53A	110.00
C2A—C1A—C6A	117.9 (6)	C31E—C51E—H53E	110.00
C2A—C1A—C11A	125.7 (5)	C31E—C51E—H51E	110.00
C6A—C1A—C11A	116.3 (5)	H51E—C51E—H53E	110.00
C2B—C1B—C6B	118.5 (7)	H52E—C51E—H53E	109.00
C2B—C1B—C11A	120.5 (6)	H51E—C51E—H52E	109.00
C6B—C1B—C11A	121.0 (6)	C31E—C51E—H52E	109.00
C1A—C2A—C3A	120.9 (6)	C2C—C1C—C6C	118.6 (6)
C1B—C2B—C3B	121.0 (7)	C6C—C1C—C11C	120.6 (5)
C2A—C3A—C4A	119.4 (6)	C2C—C1C—C11C	120.8 (6)
C2B—C3B—C4B	119.5 (8)	C2D—C1D—C11C	120.3 (5)
Br4A—C4A—C3A	120.0 (5)	C6D—C1D—C11C	120.7 (6)
Br4A—C4A—C5A	118.4 (5)	C2D—C1D—C6D	118.9 (6)
C3A—C4A—C5A	121.7 (6)	C1C—C2C—C3C	120.4 (6)
C3B—C4B—C5B	121.3 (8)	C1D—C2D—C3D	120.4 (6)
Br4B—C4B—C3B	118.6 (6)	C2C—C3C—C4C	119.7 (6)
Br4B—C4B—C5B	120.1 (6)	C2D—C3D—C4D	119.5 (7)
C4A—C5A—C6A	118.0 (6)	Br4C—C4C—C5C	119.2 (6)
C4B—C5B—C6B	119.0 (7)	C3C—C4C—C5C	121.1 (7)
C1A—C6A—C5A	122.1 (7)	Br4C—C4C—C3C	119.7 (5)
C1B—C6B—C5B	120.7 (7)	Br4D—C4D—C3D	119.7 (5)
C1A—C11A—C1B	111.1 (5)	C3D—C4D—C5D	120.8 (6)
C1A—C11A—C21A	113.8 (5)	Br4D—C4D—C5D	119.5 (5)
O11A—C11A—C21A	107.9 (5)	C4C—C5C—C6C	119.4 (7)
O11A—C11A—C1A	106.2 (5)	C4D—C5D—C6D	119.9 (6)
O11A—C11A—C1B	112.5 (5)	C1C—C6C—C5C	120.8 (6)
C1B—C11A—C21A	105.4 (5)	C1D—C6D—C5D	120.5 (7)
O21A—C21A—O22A	124.5 (6)	O11C—C11C—C1C	111.5 (5)
O21A—C21A—C11A	113.1 (5)	O11C—C11C—C1D	105.3 (4)
O22A—C21A—C11A	122.3 (6)	C1C—C11C—C1D	111.6 (5)
C41A—C31A—C51A	115.7 (16)	C1C—C11C—C21C	107.1 (5)
O21A—C31A—C41A	108.3 (12)	O11C—C11C—C21C	108.4 (5)
O21A—C31A—C51A	100.3 (12)	C1D—C11C—C21C	112.9 (5)
O21A—C31E—C41E	100 (2)	O21C—C21C—C11C	112.9 (5)
O21A—C31E—C51E	115 (2)	O22C—C21C—C11C	122.0 (5)
C41E—C31E—C51E	113 (3)	O21C—C21C—O22C	125.1 (5)
C3A—C2A—H2A	120.00	O21C—C31C—C51C	105.5 (5)
C1A—C2A—H2A	120.00	C41C—C31C—C51C	112.9 (7)
C3B—C2B—H2B	120.00	O21C—C31C—C41C	108.4 (6)
C1B—C2B—H2B	119.00	C1C—C2C—H2C	120.00
C2A—C3A—H3A	120.00	C3C—C2C—H2C	120.00
C4A—C3A—H3A	120.00	C1D—C2D—H2D	120.00
C4B—C3B—H3B	120.00	C3D—C2D—H2D	120.00
C2B—C3B—H3B	120.00	C2C—C3C—H3C	120.00
C6A—C5A—H5A	121.00	C4C—C3C—H3C	120.00
C4A—C5A—H5A	121.00	C2D—C3D—H3D	120.00
C6B—C5B—H5B	120.00	C4D—C3D—H3D	120.00
C4B—C5B—H5B	121.00	C4C—C5C—H5C	120.00
C1A—C6A—H6A	119.00	C6C—C5C—H5C	120.00
C5A—C6A—H6A	119.00	C4D—C5D—H5D	120.00
C1B—C6B—H6B	120.00	C6D—C5D—H5D	120.00
C5B—C6B—H6B	120.00	C1C—C6C—H6C	120.00
C51A—C31A—H31A	111.00	C5C—C6C—H6C	120.00
O21A—C31A—H31A	111.00	C1D—C6D—H6D	120.00
C41A—C31A—H31A	111.00	C5D—C6D—H6D	120.00
C51E—C31E—H31E	109.00	O21C—C31C—H31C	110.00
C41E—C31E—H31E	110.00	C41C—C31C—H31C	110.00
O21A—C31E—H31E	110.00	C51C—C31C—H31C	110.00
C31A—C41A—H43A	109.00	C31C—C41C—H41C	110.00
H41A—C41A—H42A	109.00	C31C—C41C—H42C	110.00
H41A—C41A—H43A	110.00	C31C—C41C—H43C	109.00
C31A—C41A—H42A	109.00	H41C—C41C—H42C	109.00
H42A—C41A—H43A	109.00	H41C—C41C—H43C	109.00
C31A—C41A—H41A	110.00	H42C—C41C—H43C	109.00
H41E—C41E—H42E	110.00	C31C—C51C—H51C	109.00
C31E—C41E—H42E	110.00	C31C—C51C—H52C	109.00
C31E—C41E—H43E	109.00	C31C—C51C—H53C	110.00
H42E—C41E—H43E	110.00	H51C—C51C—H52C	109.00
C31E—C41E—H41E	109.00	H51C—C51C—H53C	109.00
H41E—C41E—H43E	109.00	H52C—C51C—H53C	109.00
C31A—C51A—H52A	110.00		
			
C31A—O21A—C21A—O22A	5.0 (13)	C1A—C11A—C21A—O22A	−130.0 (7)
C31A—O21A—C21A—C11A	−178.7 (9)	O11A—C11A—C21A—O21A	171.2 (6)
C21A—O21A—C31A—C41A	134.8 (15)	O11A—C11A—C21A—O22A	−12.4 (9)
C21A—O21A—C31A—C51A	−103.6 (12)	C1A—C11A—C21A—O21A	53.6 (8)
C31C—O21C—C21C—O22C	10.2 (9)	C6C—C1C—C2C—C3C	−2.5 (8)
C31C—O21C—C21C—C11C	−167.4 (5)	C11C—C1C—C2C—C3C	175.8 (5)
C21C—O21C—C31C—C41C	78.6 (7)	C2C—C1C—C6C—C5C	4.0 (9)
C21C—O21C—C31C—C51C	−160.4 (6)	C11C—C1C—C6C—C5C	−174.4 (6)
C2A—C1A—C11A—O11A	−112.2 (7)	C2C—C1C—C11C—O11C	−4.6 (7)
C2A—C1A—C11A—C1B	125.2 (7)	C2C—C1C—C11C—C1D	−122.1 (5)
C2A—C1A—C11A—C21A	6.4 (10)	C2C—C1C—C11C—C21C	113.9 (6)
C6A—C1A—C2A—C3A	1.5 (11)	C6C—C1C—C11C—O11C	173.7 (5)
C11A—C1A—C2A—C3A	179.4 (7)	C6C—C1C—C11C—C1D	56.2 (7)
C2A—C1A—C6A—C5A	−1.4 (11)	C6C—C1C—C11C—C21C	−67.9 (7)
C11A—C1A—C6A—C5A	−179.4 (7)	C6D—C1D—C2D—C3D	−1.5 (9)
C6A—C1A—C11A—C21A	−175.7 (6)	C11C—C1D—C2D—C3D	−177.1 (6)
C6A—C1A—C11A—O11A	65.7 (7)	C2D—C1D—C6D—C5D	1.0 (10)
C6A—C1A—C11A—C1B	−56.9 (8)	C11C—C1D—C6D—C5D	176.5 (6)
C2B—C1B—C6B—C5B	0.4 (10)	C2D—C1D—C11C—O11C	81.2 (6)
C2B—C1B—C11A—C1A	−41.2 (8)	C2D—C1D—C11C—C1C	−157.7 (5)
C11A—C1B—C6B—C5B	−178.4 (6)	C2D—C1D—C11C—C21C	−37.0 (8)
C2B—C1B—C11A—O11A	−160.1 (6)	C6D—C1D—C11C—O11C	−94.2 (7)
C11A—C1B—C2B—C3B	179.3 (7)	C6D—C1D—C11C—C1C	26.9 (7)
C6B—C1B—C11A—C21A	−98.7 (6)	C6D—C1D—C11C—C21C	147.6 (6)
C2B—C1B—C11A—C21A	82.5 (7)	C1C—C2C—C3C—C4C	−0.8 (9)
C6B—C1B—C11A—O11A	18.7 (8)	C1D—C2D—C3D—C4D	0.6 (10)
C6B—C1B—C11A—C1A	137.6 (6)	C2C—C3C—C4C—Br4C	−176.6 (5)
C6B—C1B—C2B—C3B	0.5 (11)	C2C—C3C—C4C—C5C	2.8 (10)
C1A—C2A—C3A—C4A	−0.9 (11)	C2D—C3D—C4D—Br4D	−177.7 (5)
C1B—C2B—C3B—C4B	−1.0 (12)	C2D—C3D—C4D—C5D	1.0 (10)
C2A—C3A—C4A—Br4A	179.2 (6)	Br4C—C4C—C5C—C6C	178.0 (5)
C2A—C3A—C4A—C5A	0.1 (12)	C3C—C4C—C5C—C6C	−1.3 (10)
C2B—C3B—C4B—C5B	0.6 (12)	Br4D—C4D—C5D—C6D	177.2 (5)
C2B—C3B—C4B—Br4B	−176.4 (6)	C3D—C4D—C5D—C6D	−1.5 (10)
C3A—C4A—C5A—C6A	0.1 (12)	C4C—C5C—C6C—C1C	−2.1 (10)
Br4A—C4A—C5A—C6A	−179.1 (6)	C4D—C5D—C6D—C1D	0.5 (10)
C3B—C4B—C5B—C6B	0.3 (11)	O11C—C11C—C21C—O21C	−167.6 (5)
Br4B—C4B—C5B—C6B	177.3 (5)	O11C—C11C—C21C—O22C	14.7 (8)
C4A—C5A—C6A—C1A	0.6 (12)	C1C—C11C—C21C—O21C	71.9 (6)
C4B—C5B—C6B—C1B	−0.8 (10)	C1C—C11C—C21C—O22C	−105.7 (7)
C1B—C11A—C21A—O21A	−68.3 (7)	C1D—C11C—C21C—O21C	−51.3 (7)
C1B—C11A—C21A—O22A	108.1 (8)	C1D—C11C—C21C—O22C	131.0 (6)

### Hydrogen-bond geometry (Å, º)

**Table:**

*D*—H···*A*	*D*—H	H···*A*	*D*···*A*	*D*—H···*A*
O11*A*—H11*A*···O22*A*	0.94	2.25	2.660 (6)	105
O11*A*—H11*A*···O22*A*^i^	0.94	1.99	2.866 (6)	154
O11*C*—H11*C*···O22*C*^ii^	0.82	2.17	2.828 (6)	137
C2*A*—H2*A*···O21*A*	0.93	2.44	2.944 (9)	114
C2*C*—H2*C*···O11*C*	0.93	2.41	2.773 (9)	103
C6*A*—H6*A*···Br4*D*	0.93	2.91	3.709 (8)	144
C6*B*—H6*B*···O11*A*	0.93	2.49	2.816 (10)	101
C6*C*—H6*C*···O21*C*	0.93	2.49	2.983 (8)	113

Symmetry codes: (i) −*x*+2, −*y*+1, −*z*+1; (ii) −*x*+2, −*y*, −*z*.
